# Research on Maternal Service Area and Referral System in Hubei Province, China

**DOI:** 10.3390/ijerph19084881

**Published:** 2022-04-17

**Authors:** Lingyao Bai, Yang Cheng, Zhuolin Tao, Ling Feng, Shaoshuai Wang, Yu Zeng

**Affiliations:** 1 Faculty of Geographical Science, Beijing Normal University, No. 19, XinJieKouWai St., Haidian District, Beijing 100875, China; bailingyao@163.com (L.B.); taozhuolin@bnu.edu.cn (Z.T.); 2 Department of Obstetrics and Gynecology, Tongji Hospital, Tongji Medical College, Huazhong University of Science and Technology, 1095 Jiefang Road, Wuhan 430030, China; fltj007@163.com (L.F.); colombo2008@sina.com (S.W.); 3 Department of Obstetrics and Gynecology, West China Second University Hospital, Sichuan University, Chengdu 610041, China; zyzengyuzy@163.com

**Keywords:** hospital service area, hospital referral region, hierarchical medical system, dartmouth method

## Abstract

Hospital service area (HSA) and Hospital referral region (HRR) are significant in organizing maternal care resources in hierarchical medical systems. This quantitative study aims to delineate HAS and HRR by using obstetrics medical record data reflecting patients’ medical behavior to improve the efficiency of the utilization of medical resources. The Dartmouth method and an improved version that considers the administrative division was applied to delineate HSA and HRR by using the obstetrics medical records in Hubei Province of China in 2016. The result shows that 117 Dartmouth HSAs have a strong correlation with the county boundaries and 22 Dartmouth HRRs are highly coincident with the prefecture boundaries in Hubei. In addition, 25 improved Dartmouth HRRs within prefecture boundaries and core areas serving patients across prefecture boundaries have been identified. Based on the above results, two sets of hierarchical healthcare systems were constructed, respectively, which can provide methods and references for delineating HAS and HRR in the hierarchical medical systems in other regions of China and developing countries. The findings of this study shed light on future research and policymaking in the spatial organization of medical resources for improving the efficiency and equity in maternal care delivery.

## 1. Introduction

Improving maternal health is one of the Millennium Development Goals issued by the United Nations. The health of pregnant women in China is also an important issue in academic research and policymaking, especially since the implementation of the two-child policy in 2016 influenced the population of newborns. The births of second or later children, accounted for 45% of the newborns in 2016 [1], increasing pressure on maternal services. At present, a series of policies to promote hierarchical diagnosis and treatment have been issued by the Chinese government, these documents all highlight the significance of reasonable allocation of specialized medical resources, such as maternal services [2].

The medical systems in most countries are hierarchical, and many countries have formed medical referral systems [3]. For example, in the United States, there are three levels of hospitals, namely neighborhood health centers, general hospitals, and large medical centers. Different levels of hospitals have different responsibilities. Generally, patients need to go to community hospitals for consultation with family doctors. If necessary, patients will be referred to general hospitals or central hospitals for medical treatment [4]. Similarly, patients in general hospitals can also be referred to central hospitals. In order to reflect the healthcare utilization behavior of patients and provide reasonable units for medical resource allocation and evaluation, Dartmouth-based hierarchical medical systems have been built in many countries.

In China, the medical system is divided into three levels [5]. Primary hospitals are responsible for providing comprehensive medical services for the community, such as prevention, rehabilitation and healthcare. Secondary hospitals serve a larger spatial area, provide health services to multiple communities, receive referrals from primary hospitals, and provide technical support for treatment in primary hospitals. Tertiary hospitals are hospitals that provide medical and health services at the prefecture level or across prefectures in some cases, provide specialized medical services, solve critical and difficult diseases, and receive referrals from other hospitals. The administrative division unit is the basic unit for the allocation of medical resources, as well as the basic unit for related research [6]. So, the service area of hospitals is mostly coincidental with the administrative unit to which they belong, which is not determined based on the actual medical behavior of patients. In addition, due to the lack of a medical referral system, many patients will give priority to tertiary hospitals when it is not necessary, which may lead to a waste of medical resources [7].

Therefore, an effective and reasonable hierarchical healthcare system is of great significance. Rational and reliable hospital service areas (HSAs) and Hospital Referral Regions (HRRs) are fundamental for the construction of a hierarchical healthcare system [8]. HSA refers to the source area of most patients in the hospital, it can capture the local pattern of hospitalization. HRR refers to the space served by hospitals that receive referrals for more professional treatment [9], which is formed by the merger of HSAs. According to the Central Place Theory [10], HRR is a larger unit at a coarser geographic scale. The HSA and HRR are important references for patients to seek medical treatment at different levels. They can also be used as a research unit for evaluating the quality and efficiency of medical services and provide a management area for medical policies proposed by the government [11,12,13].

The Dartmouth method, which was created by the Dartmouth Institute for Health Policy and Clinical Practice, is frequently applied to divide HSA and HRR [8] and plays an important reference role in research on the allocation of medical resources or healthcare spending [14,15]. In recent years, with the development of spatial analysis technologies and the availability of medical records data, the method of HSA/HRR has been constantly innovated. For example, based on Huff Model Jia and Wang [9] used all discharge records of Florida in 2011 to divide HSAs in the US. Subsequently, Hu [16] applied the network optimization method to generate optimal divisions of HSAs in Florida. This method assures that the largest proportion of treatment flows are contained within HSAs while the flows between HSAs are the smallest [17]. Besides HSA and HRR, many other healthcare service areas have been developed, such as cancer service areas (CSAs) [18,19], primary care service areas (PCSAs), and [20] pediatric surgical areas (PSAs) [11].

Most studies focused on European and American countries, with few studies focusing on the construction of HSA/HRR in China. In addition, the above methods share a common feature, that is, in order to improve the degree of localized medical treatment, the HSA/HRR is different from the administrative planning boundary [21]. This is inconsistent with the reality that administrative units are the resource allocation units in China, increasing the management cost. Therefore, the applicability of such methods to construct HSA/HRR and generate a hierarchical medical system in the context of China needs to be explored.

In recent years, the Chinese government has announced a series of policies and aims to build a medical association that includes hospitals of different levels and is actively attempting to create a hierarchical medical system on this basis. However, the division of medical associations is mainly based on empirical experience and does not consider patients’ personal preferences when choosing hospitals. High-quality, high-level hospitals or specialized hospitals with outstanding professional capabilities are always identified as the headquarters of medical associations [22]. Moreover, pilot areas are generally limited within the scope of municipal units or even district units and cannot reflect feasibility in other regions. The construction of medical associations, HSA, and HRR can promote the implementation of hierarchical diagnosis and treatment. However, HSA and HRR are constructed using a large number of actual case data, better reflecting the actual medical treatment pattern than medical associations and can be better adapted to provide a reference for a wide range of medical planning.

It can be seen from the above information that most existing HSA and HRR research are conducted in developed countries. The scale and structural characteristics of the population, the distribution of medical resources, the ownership of hospitals, and the planning and allocation of medical services in developed countries are different from those in developing countries [23]. The patient register data has been regarded as the best data source to reflect patients’ personal preferences [24]. The feasibility of applying the classic Dartmouth method to construct HSA, HRR, and referral systems in developing countries needs further analysis. Therefore, this paper aims to improve the methods for identifying HSAs and HRRs and creating a referral system in China, while providing a reference for other developing countries.

Based on the above research purposes, this study takes the maternal service in Hubei Province as an example. First, the classic Dartmouth method is used to divide HSA and HRR based on the registration data of pregnant women. The HRR is jointly composed of HSAs, and the minimum service population size in each HRR conforms to the actual situation of the study area, rather than directly applying existing empirical indicators. Therefore, HSA and HRR form a two-tier referral system. In addition, according to the characteristics of China’s resource allocation based on administrative divisions as the basic unit, this paper adds the conditions of administrative division into the Dartmouth method. Dividing intra-city HRRs according to cases seeking treatment inside the city they live, and cross-city HRRs based on women seeking treatment across the city. Besides, a cross-city HRR is formed by the merger of intra-city HRRs, these two types of HRRs also meet the same minimum service population size. HSA, intra-city HRR, and cross-city HRR form a three-tier referral system. Finally, this paper compares the similarities and differences between the two referral systems, which not only provides references for China’s medical planning but also provides references for HSA and HRR research in other developing countries.

## 2. Materials and Methods

### 2.1. Study Area

**Figure 1:**
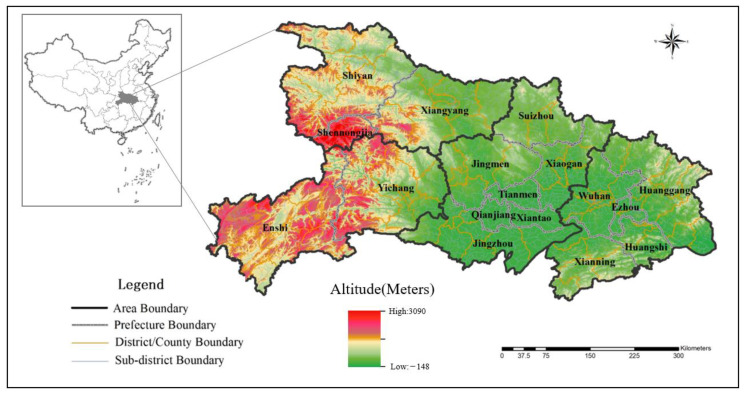
Administrative divisions, terrain, and areal division of four regions in Hubei.

Hubei Province includes 17 cities in four regions: eastern, central, western, and northern Hubei (Figure 1). The data used in this study include geographical information, attributes of hospitals, and medical records of all pregnant women in Hubei Province in 2016. The geographic information data include the administrative boundaries of cities, counties, and streets in Hubei Province. Obstetric hospital attributes, provided by Hubei Provincial Health Committee, contain information on 108 tertiary hospitals and 313 secondary hospitals and include the name, address, number of beds, doctors, nurses, and grades of each hospital. The Maternal Hospitalization records of 2016 include 660,168 pieces of data, covering 94% of the newborns in Hubei in that year. Among these records, 34.9% were recorded from tertiary hospitals, 50.8% from secondary hospitals, and 14.3% from primary hospitals. The number of obstetric beds in tertiary, secondary, and primary hospitals accounted for 22.8%, 46.9%, and 14.3% of the total beds in Hubei province, respectively. It can be seen that tertiary hospitals are the busiest, followed by secondary hospitals, these two levels of hospitals admitted most of the pregnant women in Hubei. In addition, the Hubei Provincial Health Commission advocates that secondary and tertiary hospitals offer maternal services, and most primary hospitals are not equipped with maternity beds and obstetricians. Therefore, this study focuses on the analysis of maternal resources in secondary and tertiary hospitals.

**Figure 2:**
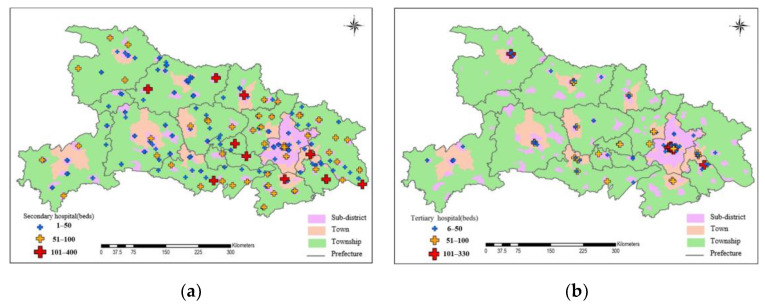
The spatial distribution of obstetrics beds in Hubei in 2016. Including: (**a**) Secondary hospitals; (**b**) Tertiary hospitals.

The coordinates of 313 secondary hospitals and 108 tertiary hospitals were obtained based on their addresses using the geocoding Application Programming Interface (API) of Baidu Map. As shown in Figure 2, secondary and tertiary hospitals are mostly distributed in towns, with more located in the eastern and central regions than in the western and northern regions. Larger secondary and tertiary hospitals are also mainly distributed in the eastern and central regions. Comparing the spatial pattern of the two levels of hospitals, it can be seen that the distribution of secondary hospitals is more uniform than that of tertiary hospitals.

### 2.2. Methods

Dartmouth method, Huff model, and network optimization method mainly aim at improving the degree of localized medical treatment ratio in the HSA\HRR. Therefore, the boundaries of HSA\HRR might be different from administrative boundaries, which is inconsistent with the Chinese policy of using the administrative divisions for healthcare resource allocation. In order to make HSA more in line with the actual situation in China, this study applies Dartmouth’s method considering the administrative divisions to identify HSAs:

(1) Hospitals were assigned to a town in which they are located, and a town with any hospital was referred to as the hospital core.
(2) Maternal flow data were spatialized, including the address of hospitals and residence sites, as well as the number of patients to hospitals.
(3) Then each town was assigned to the hospital core which serves this town most.
(4) All the towns assigned to the same hospital core constitute an original HSA
(5) Spatial adjacency, administrative divisions and majority principles (localization index greater than 0.5) were used to check and adjust the initial HSA

The meaning of the Localization index is the proportion of patients in the HSA/HRR who seek medical treatment locally [20]. It can be calculated using the following formula:

$$L_{i}=\overset{k}{\underset{j=1}{\sum }}N_{ij}/\overset{k}{\underset{j=1}{\sum }}N_{j}$$

where *L_i_* is the localized service index of the *i*-th HSA, *k* is the number of townships in the HSA, *N_ij_* is the number of patients in township *j* who choose localized medical treatment, and *N_j_* is the number of all the patients from township *j*.

**Figure 3:**
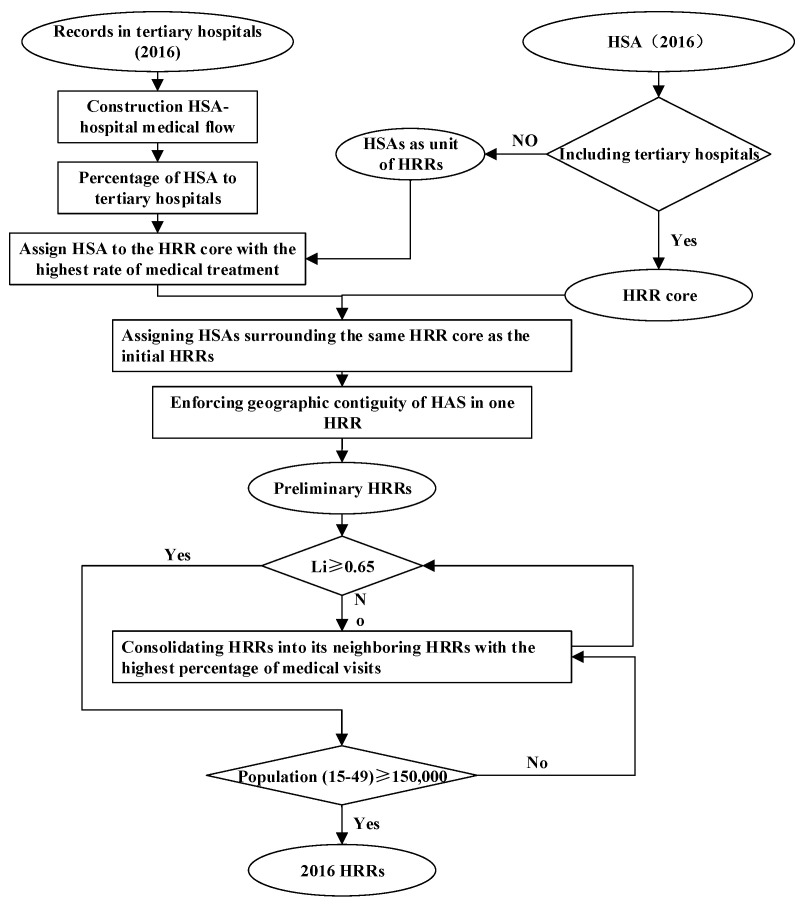
Division process of HRR.

There are three types of hospitals in China: primary hospitals, secondary hospitals, and tertiary hospitals. Among them, secondary hospitals and tertiary hospitals provide professional delivery services, with the medical resource quality of tertiary hospitals being the highest. So, in this study, secondary hospitals correspond to HSAs, and tertiary hospitals correspond to HRRs. The delineation of HRRs is similar to HSAs, however, HRRs need to meet the following conditions: The *L_i_* of each HRR is not less than 0.65 [4], and each HRR has a minimum population size, which is 120,000 in the United States. This study uses the average childbearing age population served by each tertiary hospital in Hubei as the minimum population size of each HRR because there is at least one tertiary hospital in an HRR. The specific steps are shown in Figure 3 below:

## 3. Results

### 3.1. Analysis of Maternal Hospitalization Records

**Figure 4:**
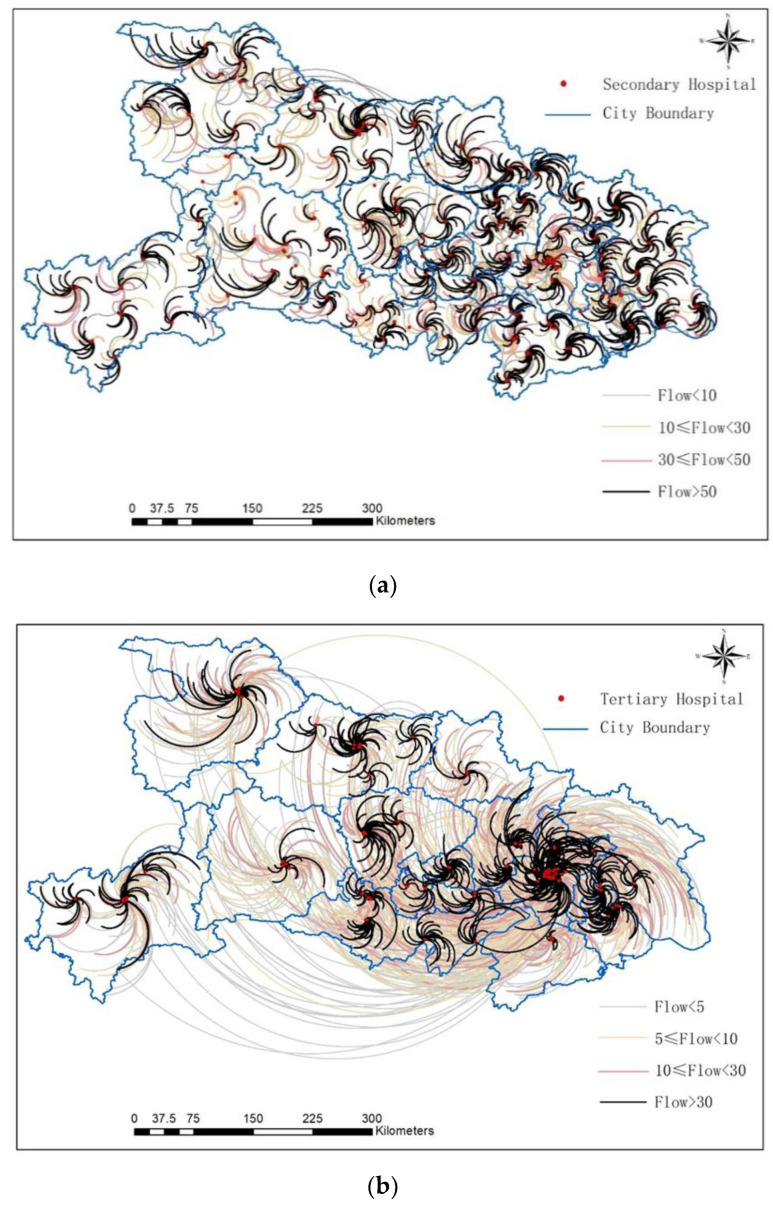
Maternal treatment flows. Including: (**a**) flows to secondary hospitals; (**b**) flows to tertiary hospitals.

The spatial and scale patterns are different between the secondary and tertiary medical flow (Figure 4). The total number of patients in the third-level medical flow is larger, but on average, each medical flow contains fewer patients than that of secondary hospitals. Among them, 335,231 parturients who went to secondary hospitals for medical treatment formed 13,128 secondary flows, with an average scale of 26, and 230,157 parturients who went to tertiary hospitals for medical treatment formed 12,793 flows, with an average scale of 18. In addition, flows point to secondary hospitals and tertiary hospitals in the western regions were mostly located in municipal administrative units, but a great number of parturients in the central and eastern regions of Hubei chose to seek medical services across city boundaries.

### 3.2. Dartmouth Based HSA

**Figure 5:**
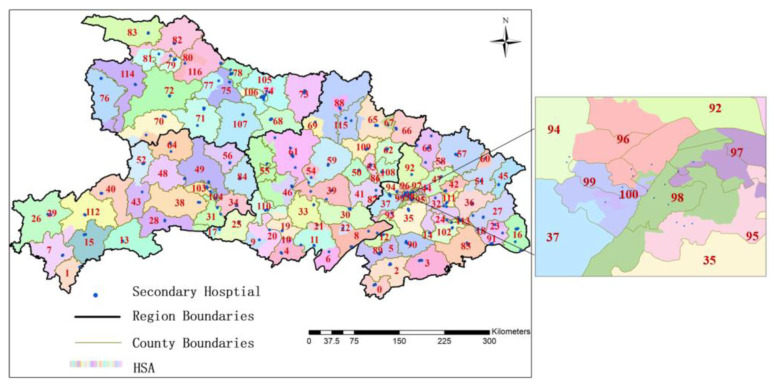
Spatial distribution of maternal HSAs. (Note: The number in Figure 5 represents HAS).

Based on the Dartmouth method, there are 117 HSAs corresponding to secondary hospitals in Hubei Province (Figure 5), including 31 HSAs with one secondary hospital, 26 HSAs with two secondary hospitals, 29 HSAs with three secondary hospitals, and 31 HSAs with four or more hospitals. On average, each medical service area has 2.7 hospitals, and each HSA serves an average of 2865 pregnant women. The *L_i_* index varies from 0.50 to 0.98. According to Figure 5 and Table 1, the number and size of HSAs in the four regions are quite different. The total number of HSAs in eastern and central Hubei is larger but the average scale is smaller, while the number and scale of medical service areas in western and northern Hubei show an opposite distribution. In addition, the boundary of HSAs has a high overlap rate with the county boundary. Most counties correspond to one HSA, and only a few districts and counties contain multiple service areas.

### 3.3. Dartmouth HRR and Administrative Dartmouth HRR

#### 3.3.1. Dartmouth HRR Based on Tertiary Medical flows

**Figure 6:**
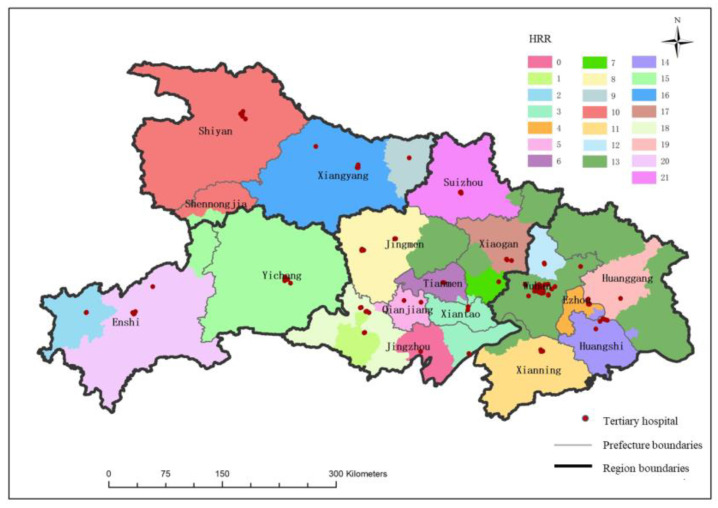
Spatial distribution of maternal HRRs.

Based on the Dartmouth HRR method, a total of 22 HRRs have been delineated in Hubei Province (Figure 6). Each HRR has an average of 4.9 hospitals. Among them, three HRRs have one tertiary hospital, five HRRs have two tertiary hospitals, five HRRs have three tertiary hospitals, and nine HRRs have four or more tertiary hospitals. The average number of patients served in each HRR is about 10,461. It can be seen from Figure 6 that there are only three HRRs in the western and northern regions of Hubei, each HRR covers a large area; there are more HRRs in the eastern and central regions of Hubei than in northern and western Hubei, the average coverage of each HRR is also relatively small. In addition to the Shennongjia forest area and the southern part of Wuhan, the boundaries of the HRRs of other tertiary hospitals overlap with city boundaries to a high degree. For example, the No. 13 HRR contains 35 tertiary hospitals in southern Wuhan. This HRR serves a large area and crosses the city boundary. It not only covers the southern part of Wuhan, but also covers some cities, such as Jingmen, Huanggang, Huangshi, and Xianning. However, in China, the administrative division unit is important for resource allocation, so if this kind of HRR is put into use it will greatly increase the management cost.

#### 3.3.2. Intra-City and Cross-City HRR

**Figure 7:**
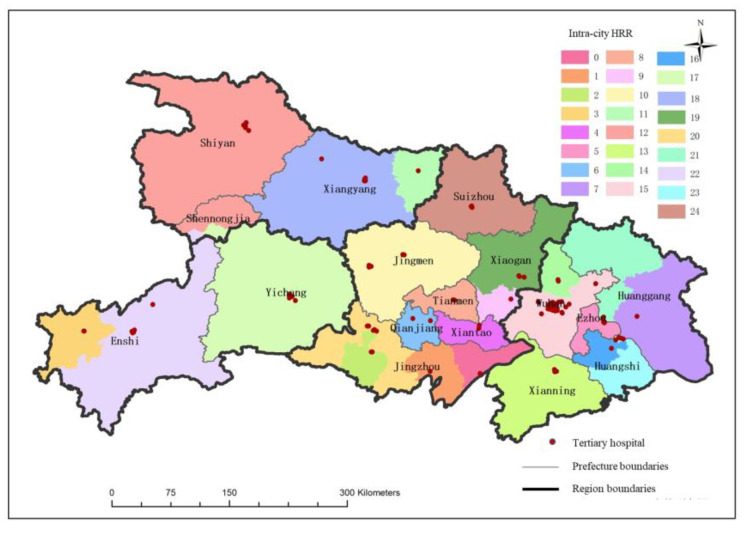
The intra-city HRR.

The above-mentioned HRRs based on all three levels of medical flows is different from the administrative unit. For example, the No. 13 HRR mainly served by Wuhan Southern Hospitals covers a large area, and for some parturients, travel cost is high. In order to construct HRRs that conform to the characteristics of China’s resource allocation, this study divides the three-level medical flow into the intra-city medical flow and cross-city medical flow, constructing the intra-city HRR (Figure 7) and the cross-city HRR based on these medical flows. Results are shown as follows:

- intra-city HRR;

According to the tertiary medical flow inside the cities, Hubei province is divided into 25 intra-city HRRs. Each intra-city HRR contains an average of 4.3 hospitals, including five HRRs with one tertiary hospital, eight HRRs with two tertiary hospitals, four HRRs with three tertiary hospitals, and eight HRRs with four or more tertiary hospitals. The spatial distribution of intra-city HRRs is similar to the classic HRR, the number of intra-city HRRs in the eastern and central regions is greater but they are smaller than those in the western and northern regions. However, the layouts of the classic HRR and intra-city HRR also have differences. The intra-city HRRs have no super-large unit like No. 13 among the classic HRRs. In addition, the boundaries of intra-city HRR are more in accordance with city boundaries. Such a distribution pattern is convenient for health management departments to supervise and manage healthcare facilities.

**Figure 8:**
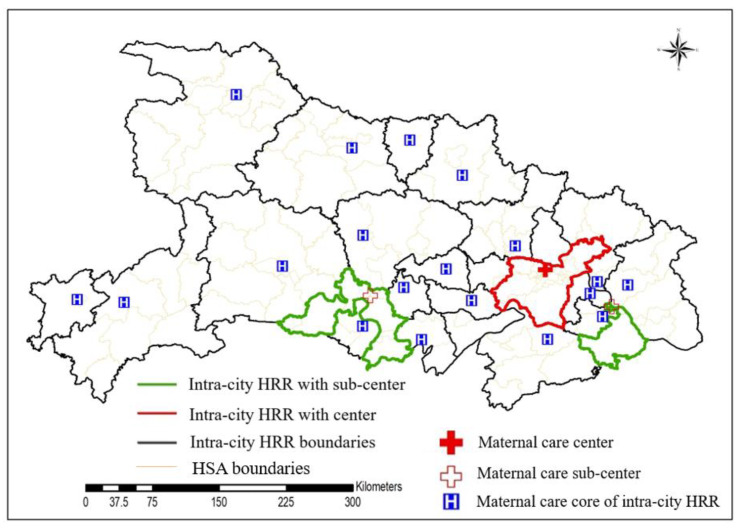
The distribution of maternal treatment center and subcenters.

- cross-city HRR;

Based on the data of 14,830 parturients using maternal services across city boundaries, this study identified the pattern of cross-city HRRs, results are shown in Figure 8. Only one cross-city HRR in Hubei province was identified, and its core area contained 35 tertiary hospitals in Wuhan. Wuhan Maternity and Child Health Hospital, Tongji Hospital, Wuhan People’s Hospital, Wuhan Union Hospital, and Wuhan University Zhongnan Hospital located in Wuhan treat more than 60% of parturients who use maternal care across cities. The intra-city HRR containing these five hospitals is the core area of the cross-city HRR and provides services for all regions of Hubei Province.

In previous studies, most hierarchical medical systems had a two-tier structure, consisting of HSAs and HRRs. This study proposes to use HSA, intra-city HRR, and cross-city HRR based on the actual maternal data of Hubei, forming a three-tier maternal hierarchical medical system. However, there is only one cross-city HRR in Hubei Province, and its core region of maternal care is the intra-city HRR in south Wuhan (Figure 8). This means, when necessary, women in remote areas of northern and western Hubei must travel more time when they are referred to Wuhan, and treatment may be delayed. The intra-city HRR in south Wuhan is the core area of the cross-city HRR. It is the most developed area with the highest density population in Hubei Province. Therefore, hospitals in this region do not only provide services for local puerperas, but also for a large number of pregnant women from other regions of Hubei, causing major pressure on maternal services.

In order to promote a more balanced development of quality maternal service, regional sub-centers were further identified in this study based on cross-city maternal data from tertiary hospitals. It was found that the intra-city HRRs codes 20 and 23, located in Jingzhou and Huangshi, are regional subcenters for parturient women who seek treatment across city boundaries. To a certain extent, the existence of these two sub-centers facilitates the medical treatment of puerpera in the west and north regions of Wuhan and relieve the pressure of obstetrics in southern Wuhan. However, there is still no regional sub-center providing maternal care services in northern and western Hubei.

### 3.4. Building the Hierarchical Medical System

#### 3.4.1. Two-Tier Hierarchical Medical System

**Figure 9:**
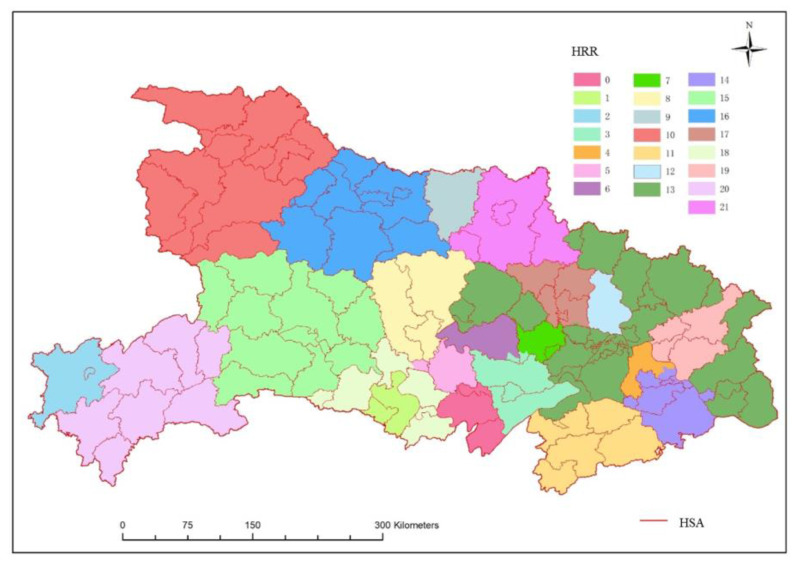
Spatial distribution map of two-tier referral system.

**Figure 10:**
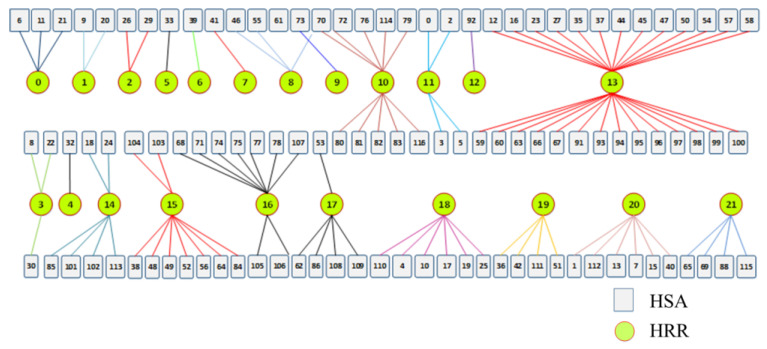
Two-tier system of referral relationship.

The two-tier maternal service referral system is composed of classic Dartmouth HRR and HSA. A total of 117 HSAs and 22 HRRs were identified in Hubei Province using the Dartmouth method, forming a total of 106 referral routes, that is, the patient can be referred to the corresponding HRR when utilizing healthcare services in HSAs within the HRR. The spatial distribution of the referral system and the referral relationship between HRR and HSA are shown in Figure 9 and Figure 10. It can be seen that the number of HSAs served by most HRRs does not exceed 10, and the 13th HRR corresponds to the most HSAs with a total of 27.

#### 3.4.2. Three-Tier Hierarchical Medical System

**Figure 11:**
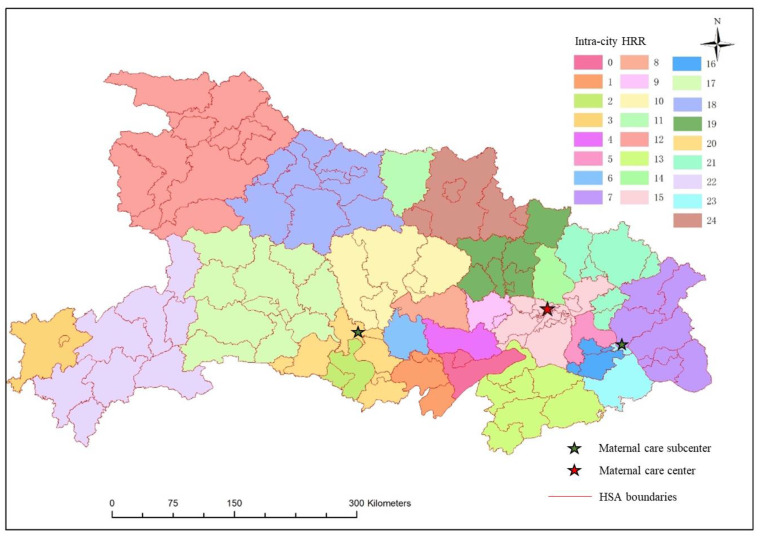
Spatial distribution map of three-tier referral system.

**Figure 12:**
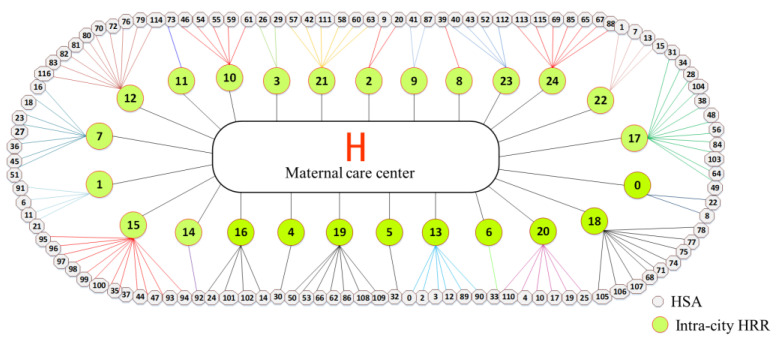
Three-tier system of referral relationship.

Considering the administrative divisions, the three-level referral system consists of HSAs, intra-city HRRs, and one cross-city HRR (Figure 11). That means, the puerperas first go to an HSA for maternal care services, and if necessary, the patients are referred to an intra-city HRR or provincial maternal service center. The patients who are referred to the intra-city HRRs can also be referred to a provincial maternal service center. This referral system has 113 referral paths between HSA and intra-city HRR and 25 paths between intra-city HRR and cross-city HRR, as shown in Figure 12. The number of HSAs corresponding to each intra-city HRR in the three-tier referral system is more than that of the two-tier system. In future planning, if the crowdedness in the maternal care center is too high, the sub-centers can be allocated refer to the locations in Figure 11.

There are two main differences when comparing the two referral systems:

The two types of referral systems have different numbers of referral routes. There are 106 referral routes from HSAs to HRRs and 113 routes from HSAs to intra-city HRRs. However, after taking cross-city HRR into consideration, referral routes are more diverse. Then the three-tier maternal service hierarchical medical system is refined. The classic HRR in the two-tier referral system crosses multiple city boundaries, and the situation is more complicated in terms of relocating medical resources. Additionally, it does not fully consider the western and northern regions with the tertiary obstetric medical resources in Hubei. The three-tier hierarchical medical system of maternal service promotes localized medical treatment and identifies the provincial center and sub-centers. This alleviates the high cost of referral travel in remote areas to a certain extent.

## 4. Discussion 

This study finds that the Dartmouth method has certain applicability in China, which can identify the actual service area of different hospitals and referral systems according to medical flow data. However, attention should be paid when applying this method in the context of China. China is a developing country with higher population mobility and more frequent changes in planning. Moreover, China mostly takes the administrative unit as the basic unit for planning and resource allocation. The disparities in healthcare quality among various regions may be larger than those in developed countries. The reference value provided by Dartmouth HSA and HRR for medical resource planning could be limited.

When Dartmouth HSA and Dartmouth HRR are used as a reference for future medical policymaking, it can ensure that all regions have a high proportion of localized medical treatment. However, HRR in developed regions only covers adjacent areas, which masks the reality that it also provides medical services for many patients in less developed regions who seek medical treatment across regions. Hospitals in cross-city HRR often need to serve both local patients and cross-city patients, which will be large in volume and may aggravate the differences in medical resources allocation among regions. Moreover, even if it is necessary, patients who choose hospitals from other cities, need to pay higher travel costs. Therefore, based on the two-tier referral system, the assessment, planning and optimization of medical resources will not provide the most reliable reference for policymaking. In addition, there is a big difference between HRR and the administrative division, so many original medical policies need to be adjusted accordingly. The professionals may also be needed to manage the medical resources in HRRs across prefectures and various HRRs’ medical resources within a prefecture.

In order to achieve equitable development of medical resources among regions in the future, medical policies should refer to the existing three-tier referral system and reflect the actual medical utilization behavior of patients to a greater extent. Even if the challenge of a large number of cross-city medical treatments cannot be reduced in the short term, the establishment of regional medical sub-centers serving cross-city medical patients, can reduce the travel cost of patients to some extent and more efficiently allocate the medical resources among regions. For example, this paper identifies the regional sub-centers located in Jingzhou and Huangshi. If these two sub-centers are fully developed, patients in remote areas from Wuhan could seek treatment across-city in less time. However, how many such sub-centers need to be set up and how many medical resources need to be allocated in different regional centers need more in-depth research.

Although the three-tier maternal service medical referral system is considered more comprehensive, this study still has some limitations: first, the division of HSA and HRR by the Dartmouth method is mainly analyzed from the perspective of the demand side. It does not consider how busy the hospitals are. Secondly, the cross-city medical treatment of parturients is affected by personal preferences and may not be necessary. Based on this division, the constructed cross-city HRR may not be the best fitting unit for tertiary hospitals. Thirdly, the tertiary obstetric resources in the southern area of Wuhan are abundant and high in quality. The different responsibilities of hospitals in this area cannot be determined in detail only through the division of HRR and HSA. Finally, the use of medical treatment flow data reflects the real medical treatment behavior and medical service utilization pattern, but the allocation of medical resources may be unequal and unreasonable in developing countries. In this context, the medical flow data may also have flaws in identifying medical service patterns.

## 5. Conclutions

This study uses actual maternal flow data and comprehensively considers the relationship between administrative boundaries and HRR boundaries for delineating HSA and HRR in the hierarchical medical system. The Dartmouth method delineated 117 Dartmouth HSAs with strong correlation with the county boundaries and 22 Dartmouth HRRs highly coincident with the prefecture boundaries in Hubei. An improved version that considers the administrative division was applied to identify 25 improved Dartmouth HRRs within prefecture boundaries and core areas serving patients across prefecture boundaries for a three-tier medical service system. The first layer is the HSA corresponding to the obstetric department of a secondary hospital, which has a high coincidence rate with the county boundary. The second level is an intra-city HRR, which has a higher rate of coincidence with the city boundary. The third level is a cross-city HRR. If the three-tier medical referral system is used as the basic unit for future medical policymaking, it can not only reflect the service pattern of secondary hospitals and tertiary hospitals to the surrounding areas, but also further depict the spatial pattern of patients’ utilization of maternal care services across cities.

These two sets of hierarchical healthcare systems were constructed, respectively, which can provide methods and references for other regions of China and developing countries. Although there are some limitations in this study, this research constructs HSA/HRR and a hierarchical medical system for China by combining the Dartmouth method and the characteristics of Chinese medical resources planning. It has enriched China’s research on the medical system and provided an important reference for the formulation of medical associations policies and hierarchical medical system policies.

## Figures and Tables

**Table 1:** Statistics of HSAs in four regions.

Region	Eastern	Middle	Western	Northern
Number of HSAs	41	35	21	20
Average area (km^2^)	1023	1492	2178	2348

## Data Availability

Restrictions apply to the availability of these data. Data was obtained from the Health Commission of Hubei, China and are available with the permission of the commission.
